# Confirmation and Fine Mapping of the Resistance Locus *Ren9* from the Grapevine Cultivar ‘Regent’

**DOI:** 10.3390/plants10010024

**Published:** 2020-12-24

**Authors:** Daniel Zendler, Reinhard Töpfer, Eva Zyprian

**Affiliations:** Julius Kühn-Institute, Institute for Grapevine Breeding Geilweilerhof, 76833 Siebeldingen, Germany; daniel.zendler@julius-kuehn.de (D.Z.); reinhard.toepfer@julius-kuehn.de (R.T.)

**Keywords:** breeding, *E. necator*, grapevine, marker-assisted selection, necrosis, powdery mildew, R-genes, *Ren9*, resistance, *V. vinifera*

## Abstract

Grapevine (*Vitis vinifera* ssp. *vinifera*) is a major fruit crop with high economic importance. Due to its susceptibility towards fungal and oomycete pathogens such as *Erysiphe necator* and *Plasmopara viticola,* the causal agents of powdery and downy mildew (PM and DM, respectively), grapevine growers annually face a major challenge in coping with shortfalls of yield caused by these diseases. Here we report the confirmation of a genetic resource for grapevine resistance breeding against PM. During the delimitation process of *Ren3* on chromosome 15 from the cultivar ‘Regent’, a second resistance-encoding region on chromosome 15 termed *Ren9* was characterized. It mediates a trailing necrosis associated with the appressoria of *E. necator* and restricts pathogen growth. In this study, we confirm this QTL in a related mapping population of ‘Regent’ × ‘Cabernet Sauvignon’. The data show that this locus is located at the upper arm of chromosome 15 between markers GF15-58 (0.15 Mb) and GF15-53 (4 Mb). The efficiency of the resistance against one of the prominent European PM isolates (EU-B) is demonstrated. Based on fine-mapping and literature knowledge we propose two possible regions of interest and supply molecular markers to follow both regions in marker-assisted selection.

## 1. Introduction

The era of accelerated plant breeding started with the emergence of marker-assisted selection (MAS). With this tool in hand, breeders dealing with woody perennials became able to select promising progeny with the desired characteristics at the very early seedling (cotyledon) stage. In grapevine, the requested characteristics are primarily resistance traits against several pathogens, as viticulture worldwide is threatened by a variety of different pests [1,2]. One of the most prominent diseases in vineyards is powdery mildew (PM) caused by the obligate biotrophic ascomycete *Erysiphe necator* (syn. *Uncinula necator* (Schw.) Burr; anamorph *Oidium tuckeri* Berk). This pathogen occurs predominantly in dry and warm regions. *E. necator* is able to grow on the surface of all green tissues of the most common cultivated grapevine *Vitis vinifera* ssp. *vinifera* (*V. vinifera*). The highest damage is caused by infection of unripe berries. At this stage, PM infection provokes the growing berries to crack open, providing entry points for any secondary bacterial and/or fungal infections eventually leading to rotting of the bunches [3,4].

Roughly 170 years ago, *E. necator* was one of the three grapevine-threats introduced to Europe by trading of grapevines derived from crosses of native North American *Vitis* species with *V. vinifera* by England, France, Spain and America [1]. This was the first encounter of *V. vinifera* with this already highly wild grapevine-adapted pathogen on the Eurasian continent, explaining the high susceptibility of the common cultivated grapevine towards PM. The combination of the pathogenic insect phylloxera (*Daktulosphaïra vitifoliae*), an obligate biotrophic oomycete causing downy mildew (*Plasmopara viticola*; DM) and spread of PM was responsible for the collapse of wine production in France and Spain roughly 150 years ago [1]. The soil borne stage of phylloxera infests the roots of grapevines causing damage and entry points for secondary infections. This results in low yield and eventually in dieback of infested grapevines after several seasons [5]. In addition, the two mildews infect all green tissues of the grapevine. Infections early in the season can lead to complete loss of harvest if DM and PM infect young flowers.

On top of these historical threats, modern viticulture faces additional annually developing fungal diseases. Two diseases caused by ascomycetes like PM are black rot (*Guignardia bidwellii* (Ellis) Viala and Ravaz; BR) and anthracnose (AN), mainly by *Elsinoë ampelina* Shear and *Colletotrichum* spp. [6]. Both diseases cause necrotic lesions on infected leaves and berries, and in both cases, infections of young developing berries, such as PM and DM, can lead to drastic reductions in yield [6].

The phylloxera problem was solved by the invention of “crafting”, the high wine-quality scions on phylloxera-resistant *Vitis* hybrid rootstocks, which is common practice in viticulture nowadays. Protection against the two mildews was achieved by the invention of the “Bordeaux mixture”, a mixture of sulphur and copper compounds that prevents the development of DM and PM when applied prior to infections [7]. This mixture was so effective that even today, 170 years later, it still plays a central role in the plant protection regime of most viticulturists, including organic wine growers. Additionally, these compounds have been proven to also inhibit the growth of AN and BR, and therefore these threats can be controlled by normal plant protection programs [6].

However, to achieve effective plant protection for the highly PM and DM susceptible *V. vinifera* cultivars, fungicides such as the sulphur and copper compounds or other synthetic protectants have to be applied, depending on the environmental conditions, up to 12 times during the growing season [8]. This makes viticulture one of the highest agricultural consumers of fungicides [9]. Furthermore, these applications make viticulture laborious and harmful for humans and the environment due to residues on grape clusters and rain wash-off from plants after treatment [10,11]. In addition, an unambiguous correlation of wine growing regions and copper accumulation in top soils was shown. This copper can be washed off into the nearby rivers and damage non-target organisms [12].

One way to reduce the enormous amounts of fungicides used in viticulture is to breed novel resistant grapevine cultivars carrying resistance traits against DM and PM combined with high wine quality [1,13]. Due to the co-evolution of DM and PM with wild *Vitis* species in North America, some accessions of these species have evolved natural genetic resistances, which either inhibit the growth of the pathogen partially or completely. In the last decades, roughly 13 of such natural genetic resistance loci against PM have been identified [14,15,16]. They reside on various chromosomes of the grapevine genome. Such loci are exploitable by grapevine breeders for introgression into new cultivars with marker-assisted selection (MAS). However, it is crucial for breeders to know which resistances to select, to achieve the most durable effect against PM. Therefore, a detailed characterization of the individual resistance loci and their function is essential. This requires inoculation experiments followed by evaluation at different time points of pathogenesis [17].

However, a reduced fungicide application may allow the other ascomycete diseases BR and AN to develop. Resistance research on these pathogens is strongly required for breeders to identify new loci for MAS to solve this potentially upcoming problem [6].

The resistance locus *Ren9* was identified during a fine-mapping study of the resistance locus *Ren3* on chromosome 15 of ‘Regent’ [16]. It is located in the anterior part of chromosome 15 spanning an interval of roughly 2.4 Mb. To confirm this locus and possibly further delimit the resistance-mediating region on chromosome 15, a cross of ‘Regent’ and ‘Cabernet Sauvignon’ was phenotypically characterized repeatedly throughout the growing season of 2016. In addition, controlled experimental inoculations were performed with selected F_1_ genotypes from that cross that carry meiotic recombinations within chromosome 15. In the frame of this work, new genetic insertion/deletion (Indel) markers were designed spanning the previously delimited region for *Ren9* with a spacing of 0.1–0.2 Mb. These markers allow a possible further delimitation of the resistance locus *Ren9* on chromosome 15 in the grapevine genome.

## 2. Results

### 2.1. Phenotypic Field-Data

**Figure 1:**
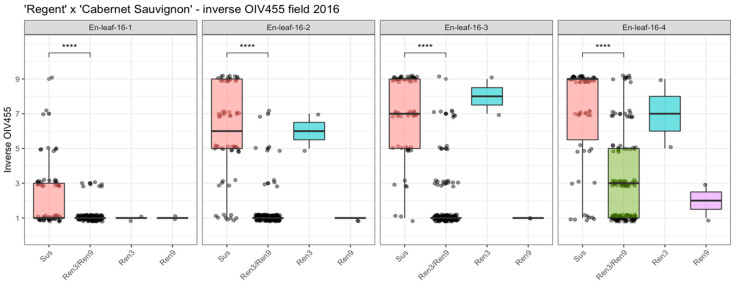
Boxplots of assigned phenotypic scores (inverse OIV455 score) for the genotypic groups of ‘Regent’ × ‘Cabernet Sauvignon’. Boxes indicate the interquartile range. The median for the respective dataset is indicated by a horizontal line in the boxplot. Points are jittered +/− 0.2 around the five classes for easier visualization of data distribution. Number of individuals: susceptible (sus) *n* = 62, *Ren3/Ren9 n* = 132, *Ren9 n* = 2, *Ren3 n* = 2. (**** = *p* ≤ 0.0001) (For data see Table S1).

Phenotypic data from the cross population of ‘Regent’ × ‘Cabernet Sauvignon’ were recorded four times during the 2016 growing season using an inverted OIV455 scale (developed from OIV, Office International de la Vigne et du Vin; International Vine and Wine Office) for reasons outlined in [16]. This approach was chosen since previous phenotypic evaluations that had been performed at the end of each season had yielded scores of around 5 to 9 for nearly all genotypes (whether they were resistant or susceptible) and were blurring genetic differences due to the late evaluation date. The same approach was applied earlier in the cross population of ‘Regent’ × ‘Lemberger’, which allowed the observation of shifting QTLs during the season [16]. According to their genotypic profiles, the F1 individuals were grouped in either resistant (*Ren3*/*Ren9*) or susceptible and individuals with either *Ren3* (“*Ren3*-only”) or *Ren9* (“*Ren9*-only”). The distribution of phenotypic data (Supplementary Materials Table S1) is visualized in Figure 1. The significance of the difference between resistant and susceptible genotypes is indicated above the boxplots (Figure 1). Differences between *Ren3*- and *Ren9*-carrying F1 individuals were not further investigated due to the fact that these two groups are represented by only two individuals each (Figure 1).

The phenotypic scores in the first scoring date were shifted towards one, as the medians indicate in the boxplots (Figure 1). The main distribution of phenotypic scores ranged from 1 to 5 in this dataset, which was due to the early date of scoring and low pathogen pressure. However, significant differences could be detected between susceptible and resistant genotypes (Figure 1, 16-1: sus—*Ren3/Ren9* ****). The median of susceptible genotypes was continuously shifted towards 9 in the 3 following datasets (Figure 1, 16-2, 16-3, 16-4). For genotypes with *Ren3*- and *Ren9*-associated alleles, the median shifted to three in the last dataset, which represented the scoring date at the end of the season with highest infection pressure (Figure 1, 16-4). The two individuals with only *Ren3* also showed a continuous shift towards a score of seven, indicating a rather strong infection with *E. necator* (Figure 1, 16-4). In contrast, for the two individuals carrying *Ren9*, the median score was shifted to a score of two at the last date (Figure 1, 16-4).

### 2.2. QTL-Analysis with Phenotypic Field-Data

**Figure 2:**
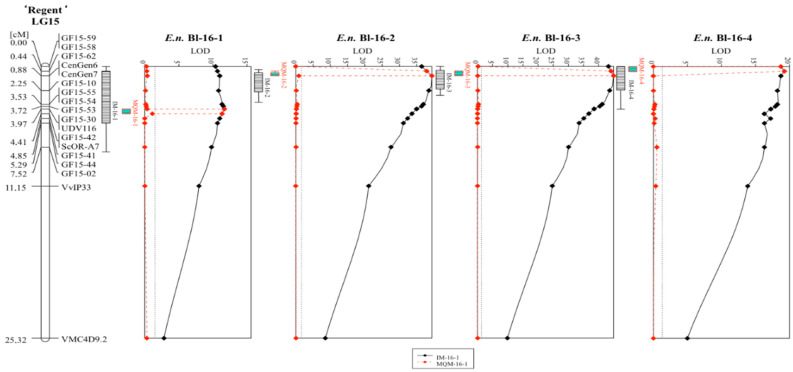
QTL graphs for the four analyzed scoring dates in 2016 with the genetic map of ‘Regent’ derived from the ‘Regent’ × ‘Cabernet Sauvignon’ mapping population. The continuous black line shows the results of IM while the dotted red line indicates the MQM results. The confidence intervals of +/−1 and +/−2 LOD values are indicated by the box and its whiskers at the left side of each graph. (IM = interval mapping, MQM = multiple QTL mapping).

The described phenotypic data were used for QTL-analysis with the previously published genetic map of ‘Regent’ × ‘Cabernet Sauvignon’ [16]. QTL analysis was performed with the maternal (‘Regent’) and paternal (‘Cabernet Sauvignon’) genetic map. Therefore, the genotypic data was coded as doubled haploid (DH) according to the manual of JoinMap^®^4.1. Results for the ‘Regent’ haplophase are listed in Table 1 and are shown as a graph in Figure 2.

The results for the ‘Cabernet Sauvignon’ haplophase are shown in Figure S1. In this haplophase, no LOD score higher than three was detected, and therefore this haplophase was not further investigated. For all scoring dates, a QTL for resistance to powdery mildew was observable on chromosome 15 (Table 1, Figure 2). The first scoring (*E.n.*-leaf-16-1) yielded a rather low LOD_max_ value of approximately 12 compared to the later three scoring dates (Table 1, Figure 2). This QTL explained around 23% of the observed phenotypic variation. The interval mapping (IM) analysis pointed to an interval spanning the region between markers GF15-62 and GF15-44 (Table 1). This represents around 8.7 Mb of chromosome 15 according to the reference genome PN40024 12X v2. The following MQM mapping limited the region to the interval around UDV116 to ScORA7 (3.1 Mb) with UDV116 being the nearest correlating marker (Table 1, Figure 2). The subsequent scoring dates yielded QTLs with LOD_max_ scores of 39 (*E.n.*-leaf-16-2) and 45 (*E.n.*-leaf-16-3) and explained up to 63% of observed phenotypic variation (Table 1). The intervals of the IM analysis were limited to CenGen6–GF15-10 for *E.n.*-leaf-16-2 (0.8 Mb) and to GF15-62–CenGen7/6 for *E.n.*-leaf-16-3 (0.2/0.5 Mb). Downstream MQM analysis limited the interval for both scoring dates to the region between GF15-62 and CenGen7/6, representing 0.2 resp. 0.5 Mb on chromosome 15 (Table 1, Figure 2). The forth scoring yielded a QTL, which was shifted completely to the beginning of chromosome 15 (Figure 2). This QTL was represented by a LOD_max_ score of 19 and represented 35% of observed phenotypic variance (Table 1). The interval of this QTL spanned the molecular markers GF15-59/58 and GF15-54/55 that corresponds to 2.7 Mb. Subsequent MQM mapping limited the interval to GF15-59/58–GF15-62 (Table 1). Taken together, a shift of the QTL from the middle part (*Ren3*) to the anterior part (*Ren9*) of chromosome 15 was observed during the time of the beginning of the season to its end.

### 2.3. Fine Mapping of the Ren9 Region in Leaf Disc Assays

Controlled infection assays were done with leaf discs from selected F1 individuals (Table 2) chosen according to their meiotic recombination points on chromosome 15.

For delimiting the region around *Ren9*, new molecular markers were designed based on insertions and deletions (Table S2). Table 2 presents the recombination points of the selected F1 genotypes from the ‘Regent’ × ‘Cabernet Sauvignon’ cross. Oligonucleotide sequences and amplicons are shown in Table S2. Individuals with *Ren3*/*Ren9* and “*Ren9*-only” showed an average inverse OIV 455 score of 2 and 1.17, respectively (moderately to highly resistant) (Table 2). Assuming the location of the resistance conferring gene of *Ren9* in the interval from CenGen7 to Indel-13, two of the recombinants showed only *Ren3*-associated alleles. These exhibited an average inverse OIV 455 score of 5.5 (moderately susceptible) (Table 2). In contrast to the two “*Ren3* only” individuals, the F1 plant 1999-074-239 showed resistance associated alleles for the markers Indel-27, Indel-23 and Indel-17 and an average phenotypic inverse OIV455 score of 1.0 (highly resistant) (Table 2).

### 2.4. Characterizing the PM Single Spore Isolate GF.En-01

For controlled infection phenotyping, leaf disc inoculation experiments were performed with the aforementioned F_1_ individuals and a single spore PM isolate, GF.En-01. The latter was sampled from a susceptible grapevine cultivar around the JKI Institute for Grapevine Breeding Geilweilerhof, Germany. Genotyping of this isolate showed that it is most likely of the EU-B type according to the identified and translated allele sizes described [18] (Table 3). There was some uncertainty for the allele sizes of EnMS-03 and -06, as they differed more than 2 bp from the published sizes (Table 3).

**Figure 3:**
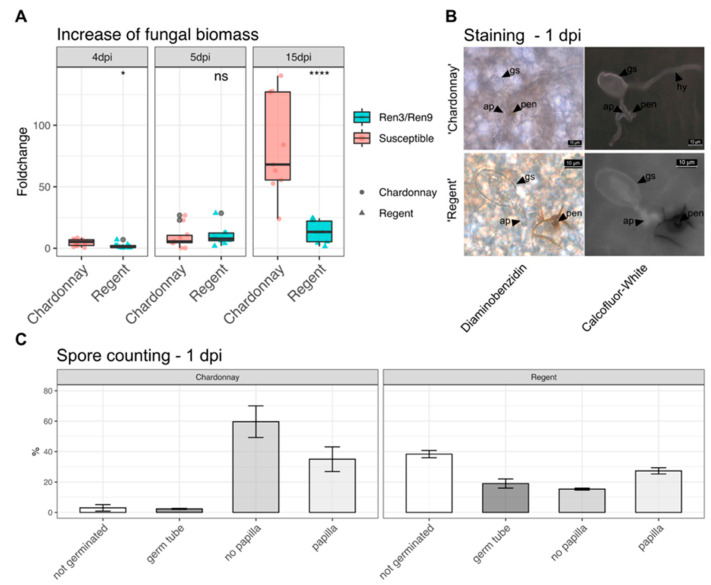
Characterization of PM isolate GF.En-01. (**A**) Fungal biomass increase over time as measured by qPCR for ‘Chardonnay’and ‘Regent’ (**** = *p* ≤ 0.0001, * = *p* ≤ 0.05, ns = not significant *p* > 0.05) (data available in Table S3). (**B**) Staining of leaves one dpi with diaminobenzidine and Calcofluor White (gs = germinated spore, hy = hyphae, ap = appressoria, pen = penetration site). (**C**) Counting of conidiospores one dpi and grouping them according to different developmental stages (data available in Table S4).

To test the aggressiveness of this isolate, inoculations with in vitro plants of ‘Regent’ and ‘Chardonnay’ were performed. Samples were taken 1, 4, 5 and 15 days post inoculation (dpi), with day one providing the reference for the latter. The increase of fungal biomass could be observed for both genotypes. At four dpi, a significant difference between ‘Regent’ and ‘Chardonnay’ was observable, which was absent at five dpi. After 15 days, a clear difference between ‘Regent’ and ‘Chardonnay’was observed with ‘Chardonnay’showing a median fold change of approximately 65 compared to a fold change of around 20 for ‘Regent’ (Figure 3A, Table S3).

In addition, at one day after inoculation, the leaf discs were stained with diaminobenzidine (DAB) and Calcofluor White (CW). DAB stain highlights reactive oxygen species (ROS) by forming a brown stain at sites with elevated ROS levels. CW stain highlights the transparent conidospores and hyphae. A clear accumulation of ROS was observable at the penetration site of the appressoria in ‘Regent’. The brown DAB stain extended around the cell in the apoplast. This reaction was much less pronounced and restricted to the actual penetration site in the susceptible ‘Chardonnay’. Furthermore, primary and secondary hyphae were observed on susceptible ‘Chardonnay’ leaves (Figure 3B).

During staining, spores were counted and grouped according to different developmental stages (Table S4). The major difference between the susceptible ‘Chardonnay’and the resistant ‘Regent’ was the overall germination rate, which was 97% in ‘Chardonnay’versus 62% in ‘Regent’. In ‘Regent’ leaves, a big portion of germinated spores showed only germ tubes at one dpi (Figure 3C). In ‘Chardonnay’, most of the spores germinated and successfully formed appressoria. In addition, no papilla formation was detectable for the biggest proportion of appressoria-forming spores (Figure 3C, ~60%). On ‘Regent’ the larger portion of germinated spores were accompanied by papilla formation (Figure 3C, ~30%). Taken together, these results indicate that *Ren3/Ren9* is capable of restricting the growth of the GF.En-01 isolate. Studying the two resistances independently should therefore be possible with this isolate. However, it indicates that *Ren3/Ren9* mediates only a partial PM resistance against this *E. necator* EU-B type isolate.

### 2.5. Leaf Disc Infection Assays with GF.En-01

Two independent inoculation experiments were performed with the single spore PM isolate GF.En-01. Datasets for hyphal growth and necrosis formation from both experiments were compared with each other in a correlation plot (Tables S5 and S6). In previous studies, a hypersensitive response (HR)/necrosis associated with the appressoria of PM was proposed as a mechanism for *Ren3*- and *Ren9*-mediated resistance [15].

**Figure 4:**
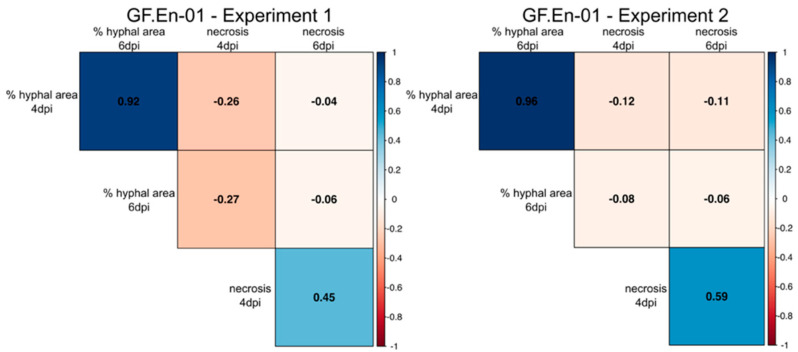
Correlation plot of the percentages of hyphal area and necrosis formation at 4 and 6 dpi. Positive correlations are indicated with blue, while negative correlations are indicated with red. The data are split into the two independent experiments. Significance level: *p* < 0.05; all correlations were significant; see Tables S5 and S6.

Here, a significant positive correlation was observed for the percentage of hyphal area present at four and six dpi comparing both experiments (Figure 4). In addition, a strong positive correlation for necrosis formation was observed for four and six dpi in both experiments (Figure 4). Percentage of hyphal area showed in all cases a negative correlation with necrosis formation. The strongest negative correlation was observed in both experiments at four dpi (Figure 4), indicating a small negative effect of necrosis formation on hyphal growth. With six dpi, only a very weak negative correlation was found between these two scored traits (Figure 4).

**Figure 5:**
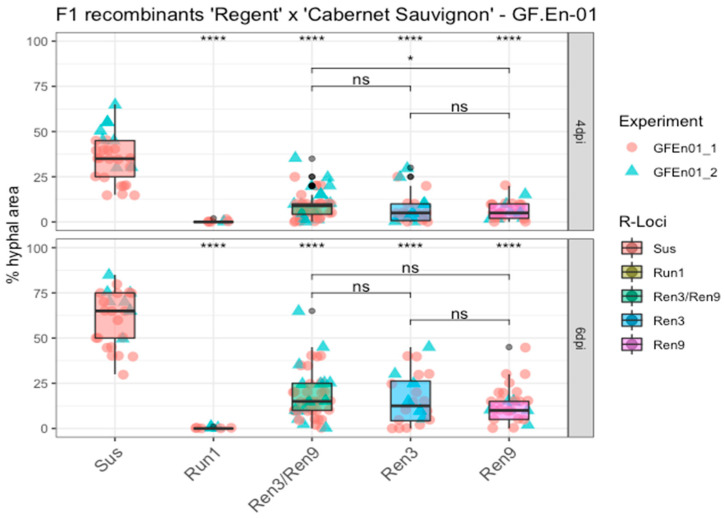
Boxplots of percentage of hyphal area at four and six dpi. F_1_ individuals with the same *R*-locus combination were grouped. Phenotypic scores from different experiments are indicated by different shapes and colors of the data points. Outliers are colored in black. Mean of respective groups are compared to the susceptible group, and the mean of the different *Ren3* and *Ren9* combinations with each other (**** = *p* ≤ 0.0001, * = *p* ≤ 0.05, ns = not significant *p* > 0.05) (Data available in Table S7).

After a global analysis of the datasets, an analysis of the different *R*-loci combinations was performed by grouping the phenotypic scores of F_1_ individuals from the ‘Regent’ × ‘Cabernet Sauvignon’ cross with similar combinations. As controls, a breeding line with the strong PM resistance locus *Run1* and the PM-susceptible genotypes ‘Cabernet Sauvignon’, ‘Chardonnay’(experiment 1, GF.En01-1) and ‘Diana’ (experiment 2, GF.En01-2) were added in the experiments (Figure 5, Sus, *Run1;* Table S7). Means of the different *R*-loci combinations were compared to the susceptible group to detect statistical differences. For all groups a significant difference with the susceptible control could be observed at four and six dpi (Figure 5). For *Run1*, a strong HR was observed, associated with the primary appressoria of the conidospores of GF.En-01, as already well documented in several studies [19,20,21] (Figure S2). This HR prevented any growth of PM on leaf discs of this genotype (Figure 5, *Run1*). In contrast to that, individuals with different *Ren3* and *Ren9* combinations showed variable resistance to PM. Phenotypic scores of *Ren3/Ren9* individuals showed the highest variation. The highest scores of the group *Ren3/Ren9* overlapped at four and six dpi, with the lowest scores belong to the susceptible control group (Figure 5).

However, the median of percentage hyphal area of the *Ren3/Ren9* group increased from approximately 12% to roughly 18%, which is a clear difference compared to the ~35% to ~65% change of the susceptible group (Figure 5). To test if there was any significant difference between “*Ren3-only*” or “*Ren9*-only” and the combination of both resistance loci, the means of these groups were compared. Only at four dpi was a significant lower percentage of hyphal area observed for “*Ren9*-only” compared to “*Ren3/Ren9*” (Figure 5). After six days, no significant differences were observed between the three groups (Figure 5).

**Figure 6:**
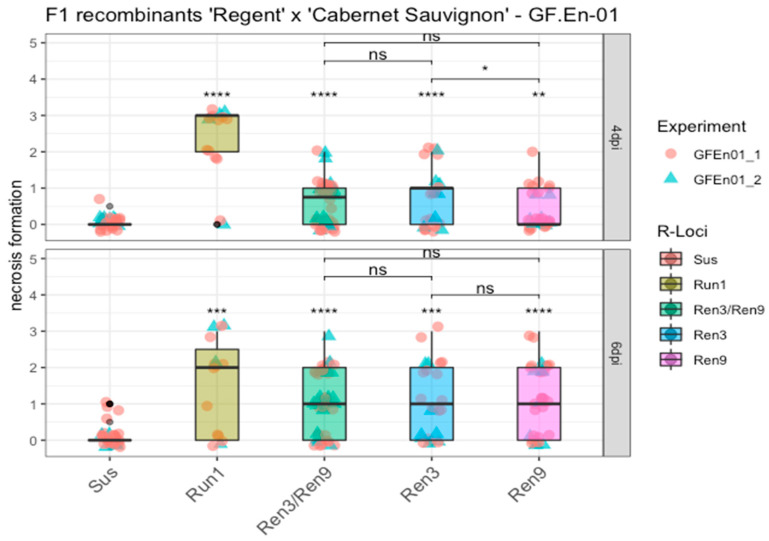
Boxplots of necrosis formation associated with appressoria at four and six dpi. F_1_ individuals with the same *R*-locus combinations were grouped. Phenotypic scores from different experiments are indicated by different shapes and colors of the data points. Outliers are colored in black. Mean of respective groups are compared to the susceptible group, and the mean of the different Ren3 and Ren9 combinations with each other (**** = *p* ≤ 0.0001, *** = *p* ≤ 0.001, ** = *p* ≤ 0.01, * = *p* ≤ 0.05, ns = not significant *p* > 0.05) (Data available in Table S7).

In addition to percentage hyphal area, trait necrosis formation was scored (Table S7). The phenotypic data were analyzed the same way, namely as percentage of hyphal area. Necrosis formation of the different *R*-loci combination carriers was compared to the susceptible control group. At both four and six dpi, the grapevines with the various *R*-loci combinations showed a significant difference compared to the susceptible group (Figure 6).

The breeding line with *Run1* showed, as already described, a strong HR associated with nearly all primary appressoria formed by the conidiospores, which is indicated by a median score of three and two at four dpi and six dpi (Figure 6, *Run1*). Median scores of *Ren3/Ren9* and “*Ren3*-only” were around one at four dpi, whereas *Ren9* showed a median score of zero at four dpi, a significant difference compared to the *Ren3* group (Figure 6). At six dpi the different combinations of *Ren3* and *Ren9* all showed a median score of one, but overall the scores ranged from zero to three (Figure 6).

## 3. Discussion

**Figure 7:**
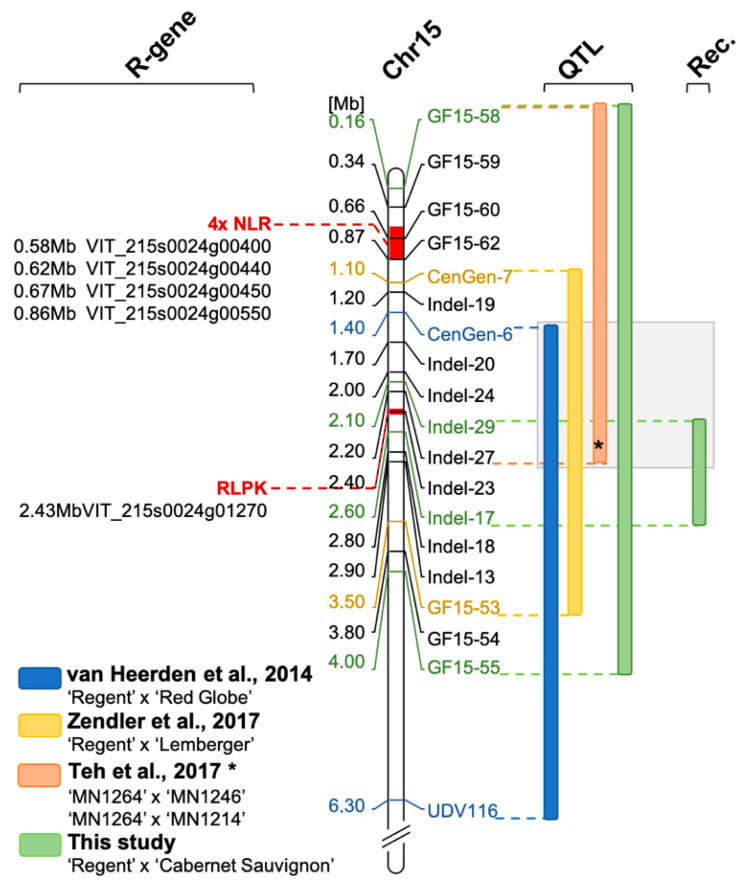
Overview of QTLs for resistance to PM in the anterior part of chromosome 15. Bars next to the map of chromosome 15 indicate QTL intervals (LOD_max_+/− 1). Physical positions are presented in respect to the reference genome of PN40024 12x v2 using the position of the molecular markers applied in this study. The QTL revealed in this study (green bar) represents the largest observed interval in the front part of chromosome 15 (enclosing the results of 16-2, -3 and -4). In addition, the interval resulting from the analysis of F_1_ individuals with meiotic recombination on chromosome 15 is indicated (Rec.). The overlap of all four QTL analyses is highlighted in grey. The region from the start of the chromosome to the position of marker GF15-55 was searched for resistance gene analogs (*R*-gene, NLR = nucleotide binding leucin rich repeat, RLPK = receptor like protein kinase). Possible regions are indicated in red. (* The physical position of the QTL interval of Teh et al. [24] was approximated to physical positions of markers in this study according to SNP positions in their supplemental material ).

Several studies reported a shift of the QTL for resistance to PM on chromosome 15. Van Heerden et al. (2014) [22] showed a LOD_max_ marker CenGen-6 associated with resistance to PM, which is located at 1.4 Mb, and a QTL interval from CenGen-6 to UDV-116 on chromosome 15 (Figure 7, blue bar). In another study, the same research team showed the *Ren3* QTL associated with marker UDV-116, which is located in the middle of chromosome 15. One could argue that if the marker density in the anterior part of chromosome 15 would have been increased, the QTL would have been possibly shifted further to the beginning of the chromosome [23]. Teh et al. [24] also investigated the resistance *Ren3* with a SNP based genetic map and phenotypic field data. Their interval for resistance to PM ranged from 0.09 to 2.2 Mb on chromosome 15 (Figure 7, orange bar).

In a previous study for the delimitation of the resistance locus *Ren3*, a second resistance associated region was identified on chromosome 15 [16]. This region, now termed *Ren9,* was located in the front part of chromosome 15 and mediated necrosis associated with the appressoria of PM at nine dpi [16]. The region of this resistance locus could be delimited to a 2.4 Mb interval flanked by the molecular markers CenGen-7 and GF15-53 (Figure 7, yellow bar) [16].

### 3.1. QTL Analysis

In the study presented here, a QTL analysis was performed with the previously published genetic map of ‘Regent’ × ‘Cabernet Sauvignon’ [16] and new phenotypic data for resistance to PM. The progression of the infections was scored at four dates during the viticulture season in 2016. Analysis after grouping the individuals into their respective *R*-loci combinations (susceptible, *Ren3, Ren9, Ren3/Ren9*) indicates a significant difference between the carriers of *Ren3/Ren9* and the susceptible group. At all four scoring dates, there is clear evidence for a positive effect of the two *R*-loci on the inhibition of PM growth (Figure 1). In the beginning of the season, the phenotypes of the majority of the genotypes were shifted towards resistance, probably due to limited natural inoculum (Figure 2, 16-1). The QTL results for this date show a QTL-region flanked by the markers UDV-116/GF15-30 and GF15-42/ScOR-A7 (Figure 2). This region agrees with the previously published localization of locus *Ren3* [16,23] and confirms it in this independent mapping study. The phenotypic scores of genotypes without any *R*-locus are shifted towards susceptible from the second scoring date onwards (Figure 1, 16-2 to 16-4) reflecting the developing PM epidemic and increasing infection pressure. The two individuals with only *Ren3* showed a median phenotypic score alike or higher than the susceptible genotypes at the later scoring dates (Figure 1, 16-2, 16-3). QTL analysis for the dates 16-2, 16-3 and 16-4 revealed a QTL shift towards the anterior end of chromosome 15 (Figure 3). The flanking markers for the QTLs of dates 16-2 and 16-3 are GF15-62 and CenGen6/CenGen7 (Figure 2, Table 1). The LOD_max_ marker in both cases is CenGen6 with a LOD score of 39.5–45 explaining 58.6–63.8% of phenotypic variance (Figure 2, Table 1). This result agrees with the finding of van Heerden et al. [25], who identified CenGen6 as the left flanking molecular marker in their QTL analysis for PM resistance. QTL analysis with the cross ‘Regent’ × ‘Lemberger’ also indicated high LOD scores for markers in the anterior part of chromosome 15 for the sampling dates 2015-1, 2015-2 and 2016-1 [16]. The interval mapping of these three dates revealed GF15-10 and CenGen-6 as left flanking molecular markers [16].

The scoring date 16-4 was at the very end of the season and the epidemic. At this time, a strong infection pressure should have been built up resulting in a shift of the phenotypic scores towards susceptibility for all carriers of R-loci (Figure 1). Yet, *Ren3/Ren9*-carrying individuals show a median score of three and most of the individuals range from one to five at this time of the season (Figure 2). The QTL analysis with this dataset shows reduced LOD scores for all markers. The QTL region, however, is still associated with the anterior region of chromosome 15. The LOD_max_ marker is GF15-62, indicating a further shift of the QTL region to the beginning of chromosome 15, in agreement with the findings of Teh et al. [24] (Figure 3, Table 1). This marker still explains about 38% of the observed variance (Table 1). Taken together, these results from four independent grapevine crosses show a high likelihood of *Ren9* being located in the front part of chromosome 15 at around 0 to 4 Mb (Figure 7). The region of overlap between all four QTLs ranges from 1.4 to 2.0 Mb, defining it as a high confidence area (Figure 7).

Additionally, the new QTL analysis presented here underscores the fact that the loci *Ren3/Ren9* mediate partial but not total resistance against powdery mildew.

### 3.2. Fine Mapping of the Ren9 Region

Detailed investigations were carried out with a subset of individuals exhibiting meiotic recombinations on chromosome 15 that separate the two resistance loci *Ren3* and *Ren9* (Table 2). Newly designed Indel markers are highlighted by a black square around them (Table 2, Table S2). The inverse OIV455 field scores from 2016 are shown, and the average was calculated (Table 2). Individuals with the same *R*-loci combination were grouped, and their phenotypic score was averaged. The combined resistance *Ren3/Ren9* or “*Ren9*-only” showed an average field inverse OIV score of ~1.2 to ~2 (highly resistant), whereas two individuals with “*Ren3*-only” showed an average inverse OIV score of 5.5 (moderately susceptible) (Table 2). Possible explanations for these results might be that during the season in 2016, a change of the composition of PM isolates took place, and the overall PM abundance increased over time. Isolates that are more virulent may emerge at the end of the season and could be capable of breaking *Ren3*. For Europe, two dominant PM isolates have been described, termed EU-A and -B [18]. Recent studies on PM isolates in vineyards in Hungary have shown EU-B to be the first isolate in the season sampled on flag-shoots. Later during the season in summer and autumn, a mixture of EU-B, -B2 and -A was detectable [26]. Similar events may happen in the vineyards around the Institute for Grapevine Breeding Geilweilerhof, Germany and would explain the results for these F_1_ individuals.

However, one individual (1999-074-239), which was previously classified as “*Ren3*-only”, showed an inverse OIV455 score of one throughout the season (Table 2). For genetic map construction, only GF15-53 (3.5 Mb) and CenGen-7 (1.1 Mb) were available as reliable molecular markers to asses recombination points in this genetic area. For fine mapping of the recombination points, new genetic Indel markers were developed in this study (Table 2, Indel, Table S2). These new molecular markers further defined the recombination points for the individuals 1999-074-239, -204 and -136 (Table 2). For the F_1_ individuals 1999-074-136 and 1999-074-204, the recombination point from susceptible to resistant was located between the markers Indel-17 (2.6 Mb) to Indel-13 (2.9 Mb). For the genotype 1999-074-239, the recombination happened between Indel-29 (2.1 Mb) and Indel-27 (2.2 Mb).

As the phenotypic scores of 1999-074-239 are similar to those of *Ren3/Ren9* and “*Ren9*-only” (Table 2, average inverse OIV score of *Ren9* = 1.2 and *Ren3/Ren9* = 2) we hypothesize that this individual is carrying both resistances. This would mean that the interval between Indel-29 and Indel-13 (2.1–2.9 Mb) could represent the *Ren9* encoding region and delimit this resistance locus to around 0.8 Mb. For breeders, this result means a much smaller introgression required to gain resistance and removal of possible genetic drag. The Indel markers designed here can easily be applied for marker-assisted selection in new breeding programs for stacking multiple resistances in novel grapevine cultivars improved in fungal resistance.

The average inverse OIV scores for the different *R*-loci combinations suggests that under field conditions of the year 2016, *Ren9* was the major resistance determinant against PM. Average inverse OIV scores of 1.2 for “*Ren9*-only” individuals and 2 for *Ren3/Ren9* plants clearly differ from “*Ren3*-only” carriers with an average score of 5.5 (Table 2). However, in a recent study, *Ren9* carrying genotypes exhibited a reduced level of resistance against PM in unsprayed fields in Italy compared to “*Ren3*-only” and *Ren3/Ren9* individuals [27]. This may indicate a different composition of PM isolates in the fields of Germany and Italy with different virulence levels breaking, respectively, the resistances encoded by *Ren3* or *Ren9*. However, this hypothesis should be treated with care. In the study presented here, the number of individuals investigated was limited, and the observations were only for one year. Further research with more individuals carrying the different *R*-loci combinations over several years is required to elucidate this observation in more detail.

### 3.3. Leaf Disc Inoculation

The individuals from Table 2 with different combinations of *Ren3* and *Ren9* were submitted to inoculation experiments with a single spore isolate sampled in the field of the JKI, Institute for Grapevine Breeding Geilweilerhof, Germany. The PM isolate GF.En-01 was genotyped with the published SSR markers [18]. The allele combinations obtained from the genotyping indicate that this isolate represents most likely the EU-B type (with some uncertainty remaining for the markers EnMS-04 and -06). This divergence can be explained by the limited precision of capillary electrophoresis and the use of different fluorescent dyes that might slightly change the apparent size of amplicons. Further, it was necessary to adapt the published sizes of Frenkel et al. [18] by subtracting the 19 bp of the M13 sequencing tag they used from the amplicon sizes obtained in capillary electrophoresis.

Growth of GF.En-01 was significantly reduced on ‘Regent’ compared to the susceptible genotype ‘Chardonnay’(Figure 3A). There is clear evidence that the development was much slower on *Ren3/Ren9* compared to the susceptible control (Figure 4C). The inhibition of growth was most likely due to the establishment of papilla and ROS at sites of penetration (Figure 3B). These are typical resistance responses against grapevine powdery mildew [14].

After this characterization, GF.En-01 was used for leaf disc inoculation experiments of the recombinant F_1_ individuals listed in Table 2. Two independent inoculation experiments were performed, yielding similar results. The data for the two traits’ percentage of hyphal area and necrosis formation were tested for correlation to investigate a possible effect of necrosis formation on hyphal growth. In the two independent experiments, a weak yet significant negative correlation between necrosis formation and hyphal growth could be observed at four dpi. This trend was much weaker at six dpi inoculation (Figure 4). However, these findings indicate that there is indeed an interaction between these traits showing that necrosis formation contributes to some extent to the inhibition of PM growth.

To investigate the effects of the different *R*-loci combinations in detail, the phenotypic scores of the individuals with similar *R*-loci were grouped. This grouping showed that there is no significant difference between the respective *Ren3* and *Ren9* combinations in terms of percentage of hyphal area covering the leaf discs except at four dpi in the comparison of *Ren3/Ren9* to “*Ren9*-only”. These two showed a significant difference with *Ren9* showing less hyphal growth. Most of the phenotypic scores are overlapping between *Ren3/Ren9* and “*Ren3*-only” making this difference marginal. Nevertheless, for all *R*-loci combinations a significant reduction in hyphal growth compared to the susceptible controls could be observed at both four and six dpi (Figure 5). These results indicate that both resistance loci by themselves are capable to detect the EU-B PM isolate and inhibit its growth. It also shows that both resistances are equally strong, and no additive effect can be observed when stacking them, at least when dealing with this specific single spore isolate.

The trait necrosis formation was investigated the same way. The *R*-loci combinations of *Ren3* and *Ren9* showed at both dates a significant difference compared to the susceptible controls. A significant difference among the combinations for “*Ren3*-only” compared to “*Ren9*-only” at four dpi inoculation was also shown (Figure 6). This might indicate that the mechanism behind the two resistances differs in terms of detection speed. This difference cannot be observed anymore at six dpi. Trailing necrosis, as it was observed for PM on leaves, is described as a part of ontogenetic resistance of grapevine berries of ‘Chardonnay’ [28,29]. However, all inoculation experiments were performed with young and healthy leaves from the shoot tip, and trailing necrosis was absent on the susceptible control leaves from ‘Cabernet Sauvignon’, ‘Chardonnay’and ‘Diana’. These results indicate that the mechanism observed here differs from the one described for ontogenetically resistant grape berries. Therefore, we propose that the resistance of *Ren3* and *Ren9* relies on a faster detection of PM pointing at specific *R*-gene interactions.

### 3.4. Possible Candidate Genes

The resistances *Ren3* and *Ren9*, although partial, might rely on different mechanisms. The resistance-associated region for *Ren3* was searched for candidate genes. This yielded a cluster of four NLR genes in the reference genome [16]. Screening the reference genome of PN40024 12x V2 in the proposed QTL interval for *Ren9* yielded two regions with *R*-gene analogs. The first region, at the very beginning between 0.5 and 0.9 Mb of chromosome 15, comprises a cluster of four possible NLR genes (Figure 7, 4xNLR) and is supported by QTLs from this study and the study of Teh et al. [24]. This cluster might look different in the genome of resistant ‘Regent’. It therefore is of high interest for further investigations. NLR genes have been proven to be key players in several plant resistance reactions against a multitude of different pathogens [30,31]. Furthermore, the well characterized resistance locus *Run1* that was used in this study as a positive control for resistance against PM was shown to rely on a NLR gene of the “Toll-Interleukin-Receptor-like” type [20].

The second region where another *R*-gene analog was found around 2.4 Mb in a region were multiple QTLs from different crosses that overlap ([16,25] and this study). In addition to the QTL intervals, the recombination-points of the F_1_ individuals 1999-074-239 and 1999-074-204 point to this region (Figure 7). The gene found here shows the typical functional domains of a leucin-rich-repeat receptor-like protein-kinase. Such functions are important for the detection of pathogen associated molecular patterns (PAMPs). One of the most prominent examples of PAMP-triggered immunity (PTI) is the detection of flagellin by the receptor-like protein-kinase (RLPK) BAK1 in a complex with other receptor-like kinases [32]. Roughly 872 of receptor-like kinases are encoded in the grapevine genome [33]. However, any important role of the RLPK gene in PM resistance has to be confirmed by functional studies. Therefore, transformations of susceptible cultivars with the possible candidate genes have to be performed, and knock-out/-down experiments with resistant cultivars carrying *Ren9* are required. If the RLPK gene would prove to be resistance conferring, this could indicate that the pathogen perception mediated by *Ren3* and *Ren9* most likely differs between the two *R*-loci. This in turn would be most interesting for breeders. A combination of different resistance mechanisms in pyramiding resistance loci is most promising to generate long-term durability.

## 4. Materials and Methods

### 4.1. Genetic Material

#### 4.1.1. Plant Material

Progeny used for genetic mapping comprised 236 F_1_ individuals from the cross of ‘Regent’ × ‘Cabernet Sauvignon’. The plants of this population are grown in the experimental fields at JKI Geilweilerhof, Siebeldingen, Germany (49°12′54.1′′ N 8°02′41.3′′ E) on their own roots with a spacing of 1.8 to 1.1 m (row by vine). They are cane pruned as is common practice in this wine growing area (Palatinate region). The plantation density at JKI Geilweilerhof, Siebeldingen, is 5050 vines per hectare. The ‘Regent’ × ‘Cabernet Sauvignon’ progeny are maintained in an experimental vineyard that was left unsprayed with fungicides. All of the 236 genotypes are represented by one plant each.

For inoculation experiments, plants were kept in the greenhouse as two eye cuttings. Plants were treated with sulphur once per week to prevent PM infection. One week prior to inoculation experiments plants were not sprayed anymore and the third fully expanded leaf, counting from the shoot tip, was sampled.

In vitro plants for inoculation experiments were contained on MS233 (Duchefa, 2.3 g/L) with sucrose (0.11 M) and gelrite (0.5% (*w*/*v*), pH 5.8) media. Plants were propagated every 12 weeks by two eye cuttings and were kept in climate chambers with 16 h light, 8 h dark and 20–22 °C.

#### 4.1.2. Powdery Mildew Isolate GF.En-01

For controlled inoculation experiments, a single spore isolate was collected from ‘Lemberger’, a susceptible grapevine cultivar grown in the fields of the Institute. The isolate was propagated every three to four weeks on surface sterilized ‘Chardonnay’leaves maintained on 1% water agar using an eyelash mounted on a Pasteur pipette. The inoculated leaves were incubated under long day conditions (16 h light, 8 h dark). Temperatures were set to 23 °C during the day and to 19 °C during the night. Around 15 days prior to inoculation experiments six surface sterilized leaves were inoculated using conidiospore chains transferred with an eyelash mounted on a Pasteur pipette.

#### 4.1.3. DNA Extraction

For DNA extraction, about 1 cm^2^ pieces of young and healthy leaves were collected from the field, the greenhouse plants or in vitro plants, transferred in plastic tubes and immediately cooled on ice upon transfer to the laboratory. Leaf segments were shock-frozen in liquid nitrogen and stored at −70 °C. DNA was extracted after grinding the samples in the frozen state with a tissue lyser mill (Retsch, 42781 Haan, Germany) using the Macherey Nagel (52355 Düren, Germany) Nucleospin 96 II DNA kit or the PeqGOLD Plant DNA mini Kit (PEQLAB GmbH, 91052 Erlangen, Germany), as described in [15].

### 4.2. Genotyping and Genetic Map Construction

#### 4.2.1. Indel Marker Design around Ren9

For the development of Indel markers in the *Ren9* region, the reference genome PN40024 12x.v2 [34,35] was used. Molecular marker development was performed, as described in Zendler et al. [16]. Sequences showing length polymorphisms greater than six bp were tested for PCR amplification. Unique flanking oligonucleotides for PCR amplification of polymorphic regions were selected according to standard conditions (~50% GC content, 20–25 bp lengths, T_a_ 55–60 °C) and are provided in Table S2. PCR reactions were performed in a 10 µL reaction mix using the Kapa 2G Multiplex Mix (PeqLAB GmbH, 91052 Erlangen, Germany) and 1 µL of 1 ng/µL diluted genomic DNA. PCR products were analyzed on 3% agarose gels with Serva Clear Stain for visualization under UV light (320 nm).

#### 4.2.2. SSR-Marker Analysis for Genetic Map Construction

The construction of genetic maps employed SSR markers. SSR marker analysis was performed in multiplex PCR assays with the Kapa 2G Multiplex Kit (PeqLAB GmbH, 91052 Erlangen, Germany) mixing up to five different oligonucleotide pairs in one PCR. The forward primer of each pair was 5′-end labeled with fluorescent dyes HEX^®^, ROX^®^, FAM^®^ or TAMRA^®^. Allele sizes were analyzed using the ABI3130XL sequencer with a 36 cm capillary set, a size standard labeled with LIZ^®^ (identical to GeneScan™ 500 LIZ™, Applied Biosystems™) and GeneMapper^®^ 5.0 software (Applied Biosystems™) [15,16].

#### 4.2.3. Construction of the Genetic Map

Genetic maps of chromosome 15 were constructed by linkage/recombination analysis using JoinMap^®^4.1 software [36]. Allele combinations observed in the SSR marker data were encoded according to the manual of JoinMap^®^4.1. The linkage group is stable up to LOD 10. Maps were calculated using the maximum likelihood mapping algorithm provided in the JoinMap^®^4.1 software. For ‘Regent’ × ‘Cabernet Sauvignon’, the previously published integrated and maternal/paternal genetic maps were used [16]. Individuals which were accidentally selfed (one individual) or had more than 30% missing data (six individuals) for the analyzed molecular markers were excluded from the mapping calculation to avoid erroneous marker order. Final analysis was based on 229 F_1_ individuals from the cross of ‘Regent’ × ‘Cabernet Sauvignon’.

### 4.3. Phenotyping

#### 4.3.1. Phenotypic Field Data

Phenotypic data for QTL analysis was obtained from evaluation of untreated field plants under natural infection pressure with *E. necator*. In former years, resistance scores had been collected once a year in late summer (end of August to end of September) following the inverse OIV (International Organization of Vine and Wine, http://www.oiv.int) classification as described [15]. In the year 2016, individuals of the cross ‘Regent’ × ‘Cabernet Sauvignon’ were scored four times every three to four weeks (26-06-16 *E.n.*-leaf-16-1, 29-07-16 *E.n.*-leaf-16-2, 18-08-16 *E.n.*-leaf-16-3, 12-10-16 *E.n.*-leaf-16-4). The degree of infection was classified in grades of 1, 3, 5, 7 and 9 (1 = no infection at all, 3 = nearly no infection visible, 5 = punctual infection spots on several leaves, 7 = punctual infection on every leaf, 9 = infections covering all leaves) (inverse to OIV descriptor 455). Each phenotypic scoring was performed by two people. Scores were assigned by visual inspection of the whole plant.

#### 4.3.2. Inoculation Experiments Using In Vitro Plants

For characterization of the *E. necator* single spore isolate GF.En-01, controlled inoculation experiments with in vitro plants of ‘Regent’ and ‘Chardonnay’were performed. Twelve leaves per genotype and sampling date were placed on 1% water agar and inoculated with a brush. Fresh conidiospores were taken from infected ‘Chardonnay’leaves on which PM was propagated (as described in 4.1.2). To characterize the development of the isolate over time, biomass increase was measured by qPCR [37]. Samples were taken at 1, 4, 5 and 15 dpi. A fold change was calculated with the delta-delta c_t_ method, using one dpi as normalization point.

Further, detailed characterization employed diaminobenzidine (DAB) and Calcofluor-White (CW) staining one dpi. Leaves were first stained by DAB according to a published protocol [38] and then exposed to CW. As the leaves of in vitro plants were very tender, the incubation time with DAB was reduced to two hours. For CW staining, leaves were treated according to the manufacturer’s instructions with one drop of CW staining solution and an equal drop of 10% KOH (Fluka chemicals). The samples were incubated for one minute and then washed with sterile water. Microscopy was performed directly after CW staining. At this point, spores were counted and grouped according to their different developmental stages. For each genotype, three times 100 spores were counted from three independent leaves.

#### 4.3.3. Leaf Disc Inoculation Experiments

For detailed investigations of selected recombinants from the ‘Regent’ × ‘Cabernet Sauvignon’ cross inoculations of leaf discs were carried out as described in [39]. In total, four leaf-discs per genotype from four different greenhouse plants were placed on 1% water agar plates. They were inoculated with a spore suspension of an *E. necator* isolate originating from a susceptible ‘Lemberger’ plant in the field.

The PM isolate GF.En-01 was grown on leaves of the susceptible cultivar ‘Chardonnay’. Around 10 to 15 days prior to the inoculation experiment six leaves from ‘Chardonnay’were surface sterilized in 1:10 diluted bleach solution (Eau de Javel, 100 mL solution containing 2.6 g NaClO) for two minutes. Leaves were rinsed three times with deionized water and dried between paper towels before they were placed in Petri dishes containing 1% water agar. Leaves were then inoculated using 10 to 15 single conidiospore chains of GF.En-01. Leaves for the inoculation experiment were surface sterilized in the same way before punching discs with a one cm diameter cork borer. The day after placing the leaf discs on 1% water agar the spore suspension was prepared by shaking the inoculated ‘Chardonnay’leaves in 15 mL sterile water with 10 µL Tween 20. Spores were counted using a hemocytometer. Spore suspensions with 1 × 10^5^–2 × 10^5^ spores/mL were used for inoculation with a pump sprayer. Visual inspection ascertained that all leaf discs were covered equally with the spore suspension. As a control the cross parental types ‘Regent’ and ‘Cabernet Sauvignon’ were included together with the susceptible cultivar ‘Chardonnay’or ‘Diana’ as well as a breeding line that carries the strong PM resistance locus *Run1* (VRH3082-1-42).

Leaf disc scoring was performed at four and six dpi. The percentage of leaf disc area covered by hyphae and necrosis formation was scored visually using a stereo microscope (Zeiss Axiozoom V16).

**Figure 8:**
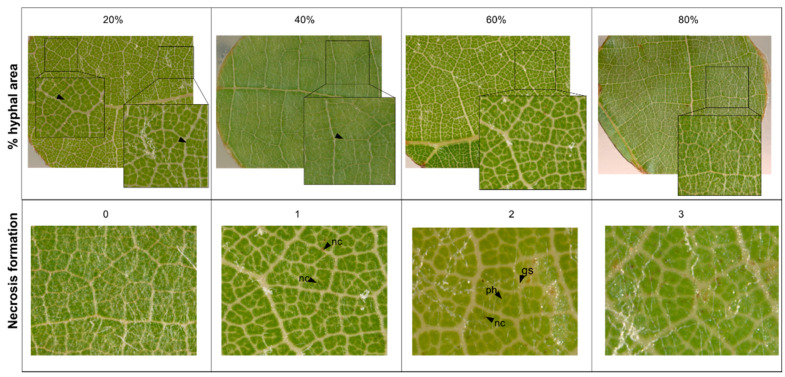
Examples for visual scoring of percentage of hyphal area- and necrosis (gs = germinated spore, ph = primary hyphae, nc = necrosis).

Necrosis formation associated with appressoria was scored on a scale of 0 to 3: 0 = no necrosis, 1 = random necrosis associated with appressoria, 2 = trailing necrosis at primary hyphae, 3 = necrosis associated with nearly all appressoria formed by PM (Figure 8). Phenotypic data was analyzed and visualized with R [40] and the packages ggpubr (stat_compare_means()) [41]. Correlations were calculated using the cor() and cor.mtest() package (method = “spearman”) and visualized with the package corrplot() of R [42]. Code and data for reproduction of graphs can be found in the Supplemental material (Tables S2–S4).

### 4.4. QTL-Analysis

QTL analysis was performed using MapQTL^®^6.0 software [43] with standard settings on the integrated and parental maps. The dataset for the separate parental maps was re-coded as doubled haploid population as recommended in the MapQTL^®^6 manual to avoid “singularity errors” [43] and enable downstream QTL analysis. The improved maternal genetic map of ‘Regent’ and the paternal genetic map of ‘Cabernet Sauvignon’ were combined with the phenotypic data from the field. Interval mapping (IM) and multiple QTL mapping (MQM) with automatic co-factor selection were performed with the datasets *E.n.*-leaf-16-1, *E.n.*-leaf-16-2, *E.n.*-leaf-16-3 and *E.n.*-leaf-16-4. A permutation test with 1000 permutations determined the linkage group (LG) specific significance threshold for each trait at *p* ≤ 0.05.

## 5. Conclusions

Both analyses from field and laboratory show that the resistance loci *Ren3* and *Ren9* mediate partial resistance to PM. QTL regions were detailed, and linked markers for application in breeding were developed. In generating new breeding lines for European viticulture, *Ren3* and *Ren9* should be complemented by strong resistance loci such as *Run1*, which completely inhibits the progression of the isolate GF.En-01 (representing the EU-B type of powdery mildew). The resistance loci *Ren3* and *Ren9* are broken in Eastern North American vineyards, as shown by inoculation experiments with NY19, an PM isolate sampled from vineyards in New York, USA [24]. In controlled inoculation experiments with this isolate, Teh and collaborators [24] could not reproduce the QTL from field data for *Ren3/Ren9*. A similar fate might await these resistance loci at some point in Europe, as the evolution of the pathogen never stops. However, the results presented here show that both resistance loci are still useful. Furthermore, the possibility of different mechanisms behind the perception of the pathogen make these resistances very interesting for breeders. With the molecular markers presented here, breeders can easily track the resistance locus *Ren9* in further breeding lines.

## Figures and Tables

**Table 1:** QTL-analysis results for powdery mildew (PM) infection severity scored at four different times of the epidemic (E.n.—leaf-16-1 to 16-4) together with the genetic map of LG15 of ‘Regent’. (IM = interval mapping, MQM = multiple QTL mapping).

	Data	Mapping	LOD _max_	% Expl	Nearest Marker	QTL-Interval (LOD_max_ ± 1)	LG15 LOD *p* ≤ 0.05	Interval [Mb]
**‘Regent’ LG15**	***E.n.*-leaf-16-1**	**IM**	11.96	23.1	GF15-30/UDV116	GF15-62–GF15-44	1.3	8.7
		**MQM**	11.96	23.1	UDV116	UDV116–ScORA7		3.1
	***E.n.*-leaf-16-2**	**IM**	39.47	58.6	CenGen6/CenGen7	CenGen6–GF15-10	1.2	0.8
		**MQM**	39.47	58.6	CenGen6	GF15-62–CenGen7/6		0.2/0.5
	***E.n.*-leaf-16-3**	**IM**	44.96	63.8	CenGen6/CenGen7	GF15-62–CenGen7/6	1.3	0.2/0.5
		**MQM**	44.96	63.8	CenGen6	GF15-62–CenGen7/6		0.2/0.5
	***E.n.*-leaf-16-4**	**IM**	19.25	34.7	GF15-62	GF15-59/58–GF15-54/55	1.2	2.7
		**MQM**	19.25	34.7	GF15-62	GF15-59/58–GF15-62		0.9

**Table 2:** Individuals from the cross ′Regent′ × ′Cabernet Sauvignon′ with meiotic recombinations on chromosome 15. SSR-markers and newly designed Indel-markers are shown: resistance associated allele (+), no resistance associated allele (−), marker not called (NA). Together with the recombination points the inverse OIV455 scorings (1—highly resistant, 9—highly susceptible) are shown. Molecular markers in regions of *Ren3* ([15], GF15-42, ScOR-A7, GF15-41) and *Ren9* (Indel-27, Indel-23, Indel-17) are marked in grey.

	**Loci**	*Ren9*	*Ren9*	*Ren9*	*Ren3/* *Ren9*	*Ren3/* *Ren9*	*Ren3/* *Ren9*	*Ren3/* *?Ren9?*	*Ren3*	*Ren3*
PN [Mb]	Marker	1999-074-068	1999-074-117	1999-074-129	1999-074-062	1999-038-017	1999-074-122	**1999-074-239**	**1999-074-204**	1999-074-136
1.1	CenGen7	+	+	+	+	+	+	−	−	−
1.2	Indel-19	+	+	+	+	+	+	−	−	−
1.7	Indel-20	+	+	+	+	+	+	−	−	−
2.0	Indel-24	+	+	+	+	+	+	−	−	−
2.1	Indel-29	+	+	+	+	+	+	−	−	−
**2.2**	Indel-27	+	+	+	+	+	+	+	−	−
**2.4**	Indel-23	+	+	+	+	+	+	+	−	−
**2.6**	Indel-17	+	+	+	+	+	+	+	−	−
2.9	Indel-13	+	+	+	+	+	+	+	+	+
3.5	GF15-53	−	**NA**	**+**	**+**	**+**	**+**	**+**	**+**	**+**
3.8	GF15-54	−	**+**	**+**	**+**	**NA**	**NA**	**+**	**NA**	**+**
4	GF15-55	−	**NA**	**+**	**+**	**+**	**+**	**+**	**NA**	**+**
6.3	UDV116	−	**+**	**+**	**+**	**+**	**NA**	**+**	**+**	**+**
7	GF15-30	−	**NA**	**+**	**+**	**+**	**NA**	**+**	**+**	**+**
**9.3**	GF15-42	−	−	−	**+**	**+**	**+**	**+**	**+**	**+**
**9.3**	ScOR A7	−	−	−	**+**	**+**	**+**	**+**	**+**	**+**
**9.6**	GF15-41	−	−	−	−	**+**	**+**	**+**	**+**	**+**
9.9	GF15-44	−	−	−	−	+	+	+	+	+
11.6	GF15-02	−	−	−	−	+	+	+	+	+
13	VvIP33	−	−	−	−	+	+	+	+	+
16.6	VMC4D9.2	−	−	−	−	+	+	+	+	+
**OIV 455**	**16-1**	1	1	1	1	1	1	1	1	1
	**16-2**	1	1	1	1	1	1	1	7	5
	**16-3**	1	NA	1	1	1	3	1	9	7
	**16-4**	3	NA	1	9	3	1	1	9	5
	**Average**	1.5 SE ± 0.5	1 SE ± 0	1 SE ± 0	3 SE ± 2	1.5 SE ± 0.5	1.5 SE ± 0.5	1	6.5 SE ± 1.9	4.5 SE ± 1.3
	**Avr./locus**	1.1667, SE ± 0.167			2, SE ± 0.34				5.5, SE ± 1	

**Table 3:** Allele sizes of the PM isolate GF.En-01 for EnMS markers [18]. Molecular markers with uncertain allele results are marked with a black box. If no allele of corresponding size was found in the list of Frenkel et al. (2012) [18], a_”?” was inserted.

	EnMS-01	EnMS-02	EnMS-03	EnMS-04	EnMS-05	EnMS-06	EnMS-07	EnMS-08	EnMS-09	EnMS-10	EnMS-11
**Allele**	219	168	218	290	167	249	177	185	155	251	171
**EU-Isolate**	A+B	B	B	B?	A+B	B?	B	B	A+B	A+B	B
**Frenkel et al., 2012**	239	185	236	305/?	186	266/?	195	205	176	271	191
**Frenkel et. al., M13 adj.**	220	166	217	286/?	167	247/?	176	186	157	252	172

## Data Availability

The data presented in this study are available in this article and the supplementary material here. Images and micrographs taken during inoculation experiments are available upon request.

## Supplementary Materials

The following are available online at https://www.mdpi.com/2223-7747/10/1/24/s1. Figure S1: QTL analysis results for the ‘Cabernet Sauvignon’ haplophase. Results for all four field-scorings are plotted in one graph. The genetic map is given on the left. Significance threshold is 1.2–1.3 as it was for the ‘Regent’ haplophase. LODmax ± 1 and 2 confidence intervals are indicated by boxes and their whiskers next to the QTL graph. Figure S2: Leaf disc of VRH3082-1-42 (*Run1*) inoculated with GF.En-01 four dpi. Magnified areas show germinated conidiospores (gs) with appressoria (ap) and a hypersensitive response (hr) associated with it. Table S1: Inverse OIV455 field scoring data used in Figure 1. Table S2: Primer sequences used to amplify the newly designed Indel-maker. Together with the primer sequences amplicon lengths and sequences of Regent (Cham = ‘Chambourcin’ haplophase, Dia = ‘Diana’ haplophase, PN40024 12xv2 and ‘Cabernet Sauvignon’ v1.1 are given. Table S3: Data used to generate the fungal biomass increase plot to characterize the growth of GF.En-01 on ‘Regent’ and ‘Chardonnay’. Table S4: Data used for spore count plot to characterize GF.En-01 on ‘Regent’ and ‘Chardonnay’. Table S5: Data used for correlation plots. This data is for the first experiment (GFEn-01_1). Use the supplied R script to reproduce the graph. Table S6: Data used for correlation plots. This data is for the first experiment (GFEn-01_2). Use the added R script to reproduce the graph. Table S7 Results of inoculations of leaf discs with PM isolate GF.En-01. This table can be used with the supplied R script to reproduce the graph.
